# (Non)canonical Wnt signaling, cytoarchitecture and stemness: new insights from primary nonmetastatic, primary metastatic, regional and distant metastatic models of adrenocortical carcinoma

**DOI:** 10.1038/s12276-025-01507-z

**Published:** 2025-11-28

**Authors:** Igor Shapiro, Huguette Debaix, Chiara Kräuchi, Andrea Abate, Stefan R. Bornstein, Svenja Nölting, Samuel Gunz, Alfredo Berruti, Sandra Sigala, Felix Beuschlein, Edlira Luca, Constanze Hantel

**Affiliations:** 1 Department of Endocrinology, Diabetology and Clinical Nutrition, University Hospital Zurich (USZ) and University of Zurich (UZH), 8091 Zurich, Switzerland; 2 Institute for Pharmaceutical Sciences, ETH Zurich, Zurich, Switzerland; 3 Section of Pharmacology, Department of Molecular and Translational Medicine, University of Brescia, Brescia, Italy; 4 Medizinische Klinik und Poliklinik III, University Hospital Carl Gustav Carus Dresden, Dresden, Germany; 5 Diabetes and Nutritional Sciences, King’s College London, London, UK; 6 Center for Regenerative Therapies, Technische Universität Dresden, Dresden, Germany; 7 Paul-Langerhans-Institute Dresden, Helmholtz Center Munich, University Hospital Carl Gustav Carus, Faculty of Medicine, Technische Universität Dresden, Dresden, Germany; 8 Lee Kong Chian School of Medicine, Nanyang Technological University, Singapore, Singapore; 9 Department of Medicine IV, University Hospital, LMU Munich, Munich, Germany; 10 Department of Computer Science, Institute for Machine Learning, ETH Zurich, Zurich, Switzerland; 11 Swiss Institute of Bioinformatics, Zurich, Switzerland; 12 Department of Molecular Life Sciences, University of Zurich, Zurich, Switzerland; 13 Medical Oncology Unit, Azienda Socio Sanitaria Territoriale Spedali Civili, Department of Medical and Surgical Specialties, Radiological Sciences, and Public Health, University of Brescia, Brescia, Italy; 14 Department of Medicine IV, LMU University Hospital, LMU Munich, Munich, Germany; 15 The LOOP Zurich-Medical Research Center, Zurich, Switzerland

**Keywords:** Adrenal tumours, Cancer models, Experimental models of disease

## Abstract

Tumor cells rearrange cytoskeletal networks, harmonize signaling pathways and modulate cell fate to initiate tumor growth and metastasis. Activating mutations in the canonical Wnt–β-catenin pathway are associated with aggressiveness and poor prognosis in adrenocortical carcinoma (ACC). However, the contribution of noncanonical Wnt pathways, which are known to regulate cell polarity and migration, is still widely unknown. Here we comprehensively investigated canonical and noncanonical Wnt signaling along with cytoskeleton networks, stemness programs and HOX gene expression in recently developed 2D and 3D cultures of primary, local and distant metastatic ACC models. We report that tumor spheroids derived from nonmetastatic primary tumors correlated strongly with the overexpression of canonical Wnt signaling components, while metastatic ACC models demonstrated shifts toward noncanonical Wnt pathways together with perturbed expression of matrix–cytoskeleton–nucleoskeleton markers and related changes in the cytoarchitecture of respective tumor spheroids. Consistent with a potential noncanonical Wnt activation in the metastatic models, an abundance and relocalization of proteins associated with tumor aggressiveness such as β-catenin, β-actin, vimentin and nucleolin as well as specific HOX-cluster activation was detected, suggesting strong potential for specific reconfigurations of metastatic tumor niches. Furthermore, all models demonstrated key properties of cancer stem cells, although with varying degrees of expression of progenitor and stem cell markers, which could be dynamically modulated by treatments and therapies. Overall, our results draw parallels between canonical and noncanonical Wnt signaling, differential cytoskeletal remodeling, stemness properties and various cellular plasticities in primary tumor and metastasis-derived models of ACC.

## Noncanonical Wnt pathways drive adrenocortical carcinoma metastasis

Almost 20 years ago, scientists discovered that Wnt signaling is often active in adrenal gland tumors. This study explores how Wnt signaling affects these tumors, especially adrenocortical carcinoma (ACC), a type of cancer with poor outcomes. Researchers focused on both canonical and noncanonical Wnt pathways. They used advanced laboratory models to examine these signaling pathways in ACC, utilizing both 2D and 3D models of ACC cells to study gene expression and protein localization. These models help mimic real tumor conditions better than traditional methods. Results showed that noncanonical Wnt signaling is linked to changes in cell structure and movement, which are important for cancer spread. The study also highlighted the role of cancer stem cell markers and HOX genes, which regulate cell growth, stemness and differentiation.

This summary was initially drafted using artificial intelligence, then revised and fact-checked by the author.

## Introduction

Almost 20 years ago, it was first reported that Wnt signaling is frequently activated in adrenocortical tumorigenesis^1^. Since then, a great deal of effort and progress has been made in our understanding of the pathophysiology of benign and malignant adrenal gland tumors. Subsequent investigations revealed that β-catenin activation is associated with a poor outcome in adrenocortical carcinoma (ACC)^2,3^ and that more than 50% of a clinically poor-outcome ACC group had *CTNNB1* (β-catenin gene) or *TP53* mutations as well as aberrant β-catenin or p53 immunostaining^4^. Further exciting studies have led to the identification of alterations in pathway-related known driver genes (such as *CTNNB1*), but also in genes not previously reported in ACC (such as *ZNRF3* or *AFF3*^5–7^). Moreover, β-catenin silencing in vitro resulted in strong antitumoral effects in the preclinical gold-standard model NCI-H295R and in the complete absence of tumor growth in vivo^8^. However, despite the considerable knowledge gained, we still lack a detailed understanding of the functionality, relevance and distinct cellular localizations of the canonical Wnt pathway—and even more so the largely unexplored noncanonical Wnt pathways—in ACC. This gap prevents a more comprehensive insight into the precise mechanisms of action of these pathways in ACC.

Much has been learned about Wnt-related factors in the stem and progenitor niche of the adrenal cortex and the multiple roles of related cell populations during the development and regeneration of the adrenal gland^9–12^. In addition, canonical and noncanonical Wnt pathways in conjunction with Notch, Hedgehog, fibroblast growth factor (FGF) and bone morphogenetic proteins (BMPs) have been implicated in the self-renewal and maintenance of cancer stem cells^13^ while the synergistic action of Wnt and HOX is involved in migration and invasiveness^14^. Hox genes regulate organogenesis throughout embryonic development, and in the adrenal gland they are believed to complement Wnt signaling. These genes contribute to the differentiation of the adrenal cortical stem cells, which maintain tissue function throughout adulthood^15^. Importantly, HOX gene expression correlates with increased proliferation, aggressive disease and poor survival of patients with ACC^16^. Furthermore, several Hox genes, including HOXB7–9, regulate matrix metalloproteases (MMPs), vimentin, E-cadherin, N-cadherin and other epithelial–mesenchymal transition (EMT) genes in migratory and metastatic cancer cells^17^. Although relevant candidates that regulate stemness, cellular plasticity and metastasis have been extensively investigated as downstream targets of Wnt and HOX signaling cascades in numerous other cancer types, little is known about their role in ACC^13,18^.

In the context of metastasization, the noncanonical Wnt signaling arms Wnt–planar cell polarity (PCP) and Wnt–Ca^2+^ are of particular interest^19^. In contrast to the canonical pathway, these signaling arms are β-catenin independent and involve, instead of canonical activators such as Wnt3A, noncanonical ligands such as Wnt5A. Interestingly, Wnt5A has been reported to antagonize canonical Wnt signaling^20,21^. Another ligand, Wnt4, has been demonstrated to regulate either β-catenin-dependent or β-catenin-independent signaling and can either activate or suppress different signaling arms^22^. The Wnt–PCP signaling pathway, for example, can be activated downstream of Wnt5A, ROR1/2, Vangl, Lgr4/5, related FZD receptors and Dishevelled (DVL) adaptor proteins leading to the activation of RhoA, ROCK and c-Jun-N-terminal kinase (JNK) signaling. Ultimately, these signaling molecules stimulate actin polymerization, reorganize the cytoskeleton and coordinate cellular orientation, thereby dictating polarized cell morphology and directional cell movements during invasion and metastasization of cancers^23^. The noncanonical Wnt–Ca^2+^-pathway shares some candidates with Wnt–PCP such as Wnt5A or ROR1/2. In contrast to Wnt–PCP, however, Wnt–Ca^2+^ signaling induces Ca^2+^ release from the endoplasmic reticulum membrane and activates protein kinase C and Cdc42, subsequently leading to actin polymerization and the induction of transcription factors of the NFAT-family, which together contribute to cell polarization and migration^23^. The reorganization of the cytoskeleton is pivotal to migration because actin and vimentin underlie the formation of invadopodias, the specialized protrusions on the leading edge of cells used to perforate the basement membrane by cells with metastatic potential^24^. Dynamic actin and vimentin networks characterize the invadopodias, along with integrins, glycoproteins such as CD44, and MMPs^24^. Signaling molecules including RhoA and Cdc42 as well as transcription factors downstream of noncanonical Wnt orchestrate many of the steps in invadopodia biogenesis^24^.

In the current study, we aim to extend the preclinical knowledge and toolbox to gain further insights into the cell biological aspects of Wnt signaling and related cellular plasticities in ACC. Therefore, we used recently developed two-dimensional (2D) and three-dimensional (3D) models of primary (NCI-H295R), regionally metastatic (TVBF-7) and distant metastatic (MUC-1) ACC and comprehensively compared candidates related to Wnt signaling, cytoskeleton (such as actin, intermediate filaments and related components of the nuclear lamina), EMT and stemness. Our study reveals hybrid EMT and Wnt stages for the metastatic models TVBF-7 and MUC-1, including shifts toward noncanonical Wnt signaling (Wnt–Ca^2+^ and Wnt–PCP, respectively). Consistent with the distinct programs, we detect specific localization of noncanonical Wnt proteins, related cytoskeletal rearrangements, loss of apical-to-basal cell polarity, EMT markers and chromatin remodeling, with the most pronounced phenotype in the model representing Wnt–PCP signaling, MUC-1. The investigation of a broad panel of stem cell markers and HOX clusters revealed clear evidence for the abundance of a wide variety of classical stem cell markers (such as CD44, CD24 and ALDH1). Finally, specific activities of HOX genes were detected, including exclusive activation of HoxD10 in Wnt5-overexpressing metastatic models and a subsequent correlation analysis confirming a link between Wnt5A and HOXD10 expression in clinical patient samples of ACC.

## Material and methods

### RNA sequencing

ACC cell lines were plated in six-well plates (TPP) at an appropriate density and exposed to vehicle or treated with different stimuli as described in ref. ^25^. Cells were subsequently incubated for 1 day. RNA was extracted with an RNeasy Mini kit (Qiagen) and treated with DNAse (TurboDNAfree kit, Thermo Fisher Scientific) following the manufacturer’s protocols. RNA sequencing was performed by the Genomics Facility Basel, Department of Biosystems Science and Engineering (D-BSSE), Basel, Switzerland. Information about the bioinformatics workflow can be found in ref. ^25^.

### 2D and 3D spheroid culture

Human ACC cell lines, namely NCI-H295R, MUC-1 and TVBF-7 cells, were basally cultured as indicated elsewhere^26,27^. Detailed information about 2D culture conditions including specific compounds, cell numbers, incubation times and statistical analysis can be found in ref. ^25^. A human primary culture of a primary ACC at an advanced, metastasized stage was established as previously reported^26^. Tumor sample collection was approved by the local ethics committee (Kantonale Ethikkommission Zürich, BASEC 2017-00771), and written informed consent was obtained before tumor sampling.

To obtain spheroids, ACC cells were plated in spherical plates 5D (Kugelmeiers) for 14 days at an appropriate density, depending on cell type^28^. Spheroids were fixed in 10% phosphate-buffered formalin, paraffin-embedded (Especialidades Médicas MYR, Tarragona, Spain), and 4-µm sections were subsequently prepared using a Leica RM2255 microtome (Leica Biosystems).

### Immunofluorescence

Sections were deparaffinized, followed by heat-induced epitope retrieval (HIER) treatment in sodium citrate buffer, then blocked and incubated overnight at 4 °C or for 2 h at room temperature with the primary antibody. Subsequently, the secondary antibody was applied at room temperature for 1 h. At the end of the incubation, in the case of multiple staining, another primary antibody was applied with the same procedure followed by the appropriate secondary antibody. DAPI was added (final concentration 1 µg/ml) during the incubation with the last secondary antibody. In Table 1, primary and secondary antibodies are listed. Pictures were acquired with a Zeiss Axio Imager M2 equipped with an AxioCam 512 color camera, using ZEN 3.3 software (Carl Zeiss).

**Table 1:** Complete list of antibodies used for the described experiments.

Antibody	Catalog number, company	Dilution
FOXA1	ab170933, Abcam (Cambridge, UK)	1/100
CD44	3570, Cell signaling (Danvers, MA, USA)	1/100
Nestin	73349, Cell Signaling (Danvers, MA, USA)	1/800
Nanog	4903, Cell Signaling (Danvers, MA, USA)	1/200
CD68	ab955, Abcam (Cambridge, UK)	1/100
CD133	64326, Cell Signaling (Danvers, MA, USA)	1/100
ALDH1A1	ab52492, Abcam (Cambridge, UK)	1/200
CD24	67627-1, Proteintech (Manchester, UK)	1/800
KLF4	MA5-41214, Invitrogen (Waltham, MA, USA)	1/200
Oct-4	MAA424Hu21, Cloud-Clone Corp. (Katy, TX, USA)	1/100
ALDH1A3	CSB-PA001567LA01HU, Cusabio (Houston, TX, USA)	1/200
SOX9	CSB-RA202969A0HU, Cusabio (Houston, TX, USA)	1/200
β-Catenin	610153, BD Biosciences (NJ, USA)	1/250
Vimentin	abx431705, Abbexa (Cambridge, UK)	1/100
WNT5A	MA5-41244, Invitrogen (Waltham, MA, USA)	1/50
N-cadherin	abx123447, Abbexa (Cambridge, UK)	1/150
E-cadherin	abx002199, Abbexa (Cambridge, UK)	1/150
SOX2	CSB-PA16539A0Rb, Cusabio (Houston, TX, USA)	1/150
MYC	CSB-PA407491, Cusabio (Houston, TX, USA)	1/50
NR5A1 (SF-1)	KAL-KO611, Cosmo Bio Co (Carlsbad, CA, USA)	1/100
LaminB1	Sc-365214, SANTA CRUZ Biotech (Dallas, TX, USA)	1/100
Nucleolin	STJ140144, St John’s Laboratory (London, UK)	1/200
β-Actin	A-5441, SIGMA-ALDRICH (St. Louis, MO, USA)	1/100
Actin	A2066, SIGMA-ALDRICH (St. Louis, MO, USA)	1/50
MMP9	AF911, R&D systems (Minneapolis, MN, USA)	1/100
Wnt4	CSB-PA026137LA01HU, Cusabio (Houston, TX, USA)	1/100
Donkey anti-goat Alexa 568	A11057, Invitrogen (Waltham, MA, USA)	1/500
Goat anti-mouse Alexa 488	A21121, Invitrogen (Waltham, MA, USA)	1/500
Goat anti-mouse Alexa 568	A11004, Invitrogen (Waltham, MA, USA)	1/500
Donkey anti-mouse Alexa 647	A32787, Invitrogen (Waltham, MA, USA)	1/200
Goat anti-rabbit Alexa 488	A11034, Invitrogen (Waltham, MA, USA)	1/500

### Immunohistochemistry

To evaluate the morphology of spheroids, sections were immunohistochemcally stained and counterstained with hematoxylin–eosin or methylene green as previously described^28^. Sections were deparaffinized, rehydrated, treated with heat-induced epitope retrieval and incubated in blocking buffer containing 3% bovine serum albumin (Roche Diagnostics), 5% goat serum (Jackson ImmunoResearch Laboratories) and 0.5% Tween 20 (Sigma-Aldrich). Samples were incubated overnight at 4 °C with the β-catenin primary antibody. The secondary antibody was applied at room temperature for 1 h. Primary and secondary antibodies are listed in Table 1. After incubation with the ABC solution (Vectastain Elite ABC-HRP Kit, Vector Laboratories), bound antibodies were visualized by 3,3′-diaminobenzidine staining. Hematoxylin counterstaining was carried out by incubating the slides with Harris HTX Hematoxylin (HistoLab), followed by the differentiation solution described above. Subsequently, the samples were dehydrated and mounted with Pertex mounting medium (HistoLab). Pictures were acquired with a Zeiss Axio Imager M2 equipped with an AxioCam 512 color camera, using ZEN 3.3 software (Carl Zeiss, Oberkochen, Germany).

### Bioinformatical and statistical analysis

The results shown here for the correlation of Wnt5A with HOXD10 in ACC clinical patient samples are based upon data generated by the The Cancer Genome Atlas (TCGA) Research Network (https://www.cancer.gov/tcga). Further data presentation and statistical analysis were performed using GraphPad Prism, with either a classical heatmap or one-way analysis of variance followed by Dunnett’s post-test (*n* = 3 biological replicates). Further bioinformatical analysis was performed as described in detail previously^25^.

## Results

First, we compared the gene expression of a broad panel of Wnt-relevant candidates in ACC models at basal conditions and following different stimuli. We implemented three well-characterized human adrenocortical cell lines that provide specific characteristics^25,27^: NCI-H295R had been obtained from a primary tumor, exhibits steroidogenic overactivation, but retains activation by physiological stimulators. This cell line has also been regarded as a responder to a number of therapeutic agents also used or tested in clinical practice. TVBF-7 is derived from a regional metastasis, has been demonstrated to show autonomous cortisol production and is characterized as partially drug resistant. MUC-1 had been obtained from a distant metastasis and has been demonstrated to retain basal steroidogenic properties under basal 2D conditions. In addition, this cell line has been shown to be highly drug resistant. Recently, we characterized the super-enhancer landscape of ACC and closely matched these cell lines with patient samples and clinically relevant ACC cluster types C1A (SF-1-high, steroidogenic-high/TVBF-7 and NCI-H295R) and C1B (SF-1, steroidogenic-low/MUC-1). Thus, in sum, the models reflect preclinically relevant heterogeneous clinical conditions^25,27^. Interestingly, our previous study revealed also a remarkable dynamic shift in the main cluster types when treated with epigenetic compounds JQ1 and THZ-1, as well as the differentiation enhancer forskolin and the adrenolytic drug mitotane. These findings uncovered an overall unexpected high cellular plasticity with potentially dynamic modulation of cell fate decisions^25^. Accordingly, we started our investigations of Wnt-related candidates under the same cell-fate-modulating conditions.

### Gene expression of canonical and noncanonical Wnt components

**Fig. 1:**
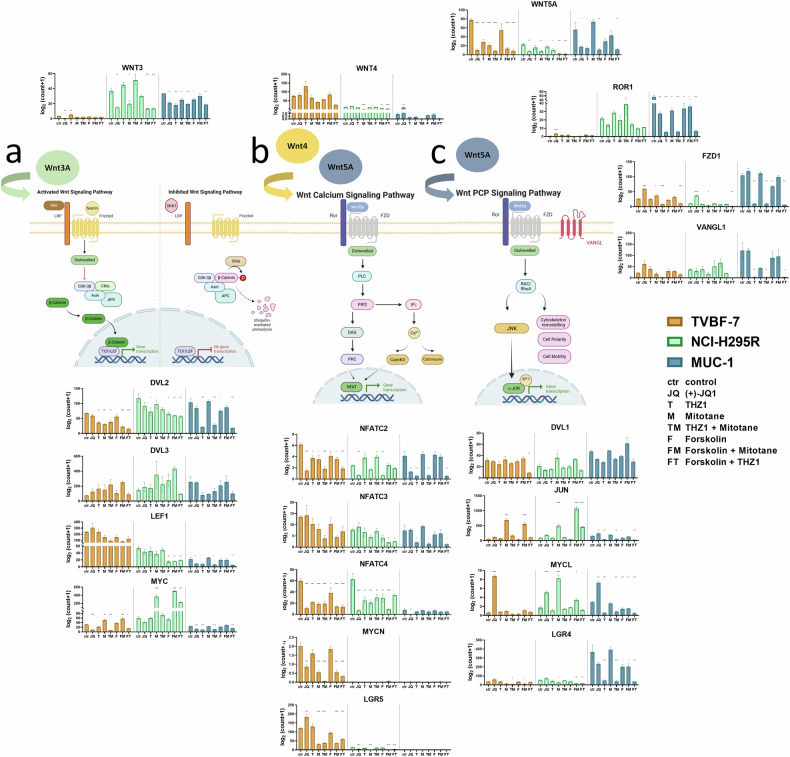
Dynamic modulation of canonical and noncanonical Wnt signaling. **a**–**c** Schematic depictions of Wnt–β-catenin (**a**), Wnt–Ca^2+^ (**b**), Wnt–PCP (**c**) and the expression of selected candidates belonging to these different pathway arms. Pathways and their related ligands (such as canonical Wnt3A, noncanonical ligands Wnt4 and Wnt5A, and noncanonical receptors such as ROR1 or VANGL1) or side or downstream effectors such as transcription factors are presented close to the respective canonical or noncanonical pathways arms. TVBF-7, NCI-H295R and MUC-1 are colored in orange, turquoise and blue, respectively. Drug treatments are depicted from left to right for each model as untreated controls (C), JQ1 (J), THZ-1 (T), mitotane (M), TM, forskolin (F), FM and FT (*n* = 3). All graphs are again presented separately and in higher magnifications in Supplementary Figs. 1 and 2 to provide optimal readability for each cell line, drug and condition. Created with BioRender.com.

We investigated the activation of the canonical Wnt–β-catenin axis in all models under these conditions, which revealed a peak in the expression of WNT3A, DVL2 and MYC in NCI-H295R (Fig. 1a). Moreover, we found a decline in the expression of some Wnt components such as LEF-1 or Wnt4, correlating with the expression of SF-1 in the three models, such that TVBF-7 has the highest expression of SF-1/Wnt and MUC-1 the lowest. Interestingly, TVBF-7 and MUC-1 demonstrated in addition shifts toward the noncanonical Wnt–Ca^2+^ (TVBF-7: WNT5A, LGR5 and NFATs) and Wnt–PCP (MUC-1: WNT5A, VANGL1, ROR1 and LGR4) signaling arms, respectively (Figs. 1b,c). Overall, we repeatedly detected during our analysis two types of dynamics in gene expression levels: amplitudes over the models representing the three Wnt-signaling arms (Wnt–Ca^2+^, Wnt–β-Cat and Wnt–PCP) and oscillations within each model depending on the specific stimulation. Importantly, expression of Wnt components oscillated strongly upon stimulation, showing a tendency to switch toward each other’s expression levels, as exemplified for ROR1, LGR4, MYCL, Wnt3 and JUN upon THZ-1 treatment and Wnt5A upon mitotane treatment.

#### Cellular localizations of canonical and noncanonical Wnt components

**Fig. 2:**
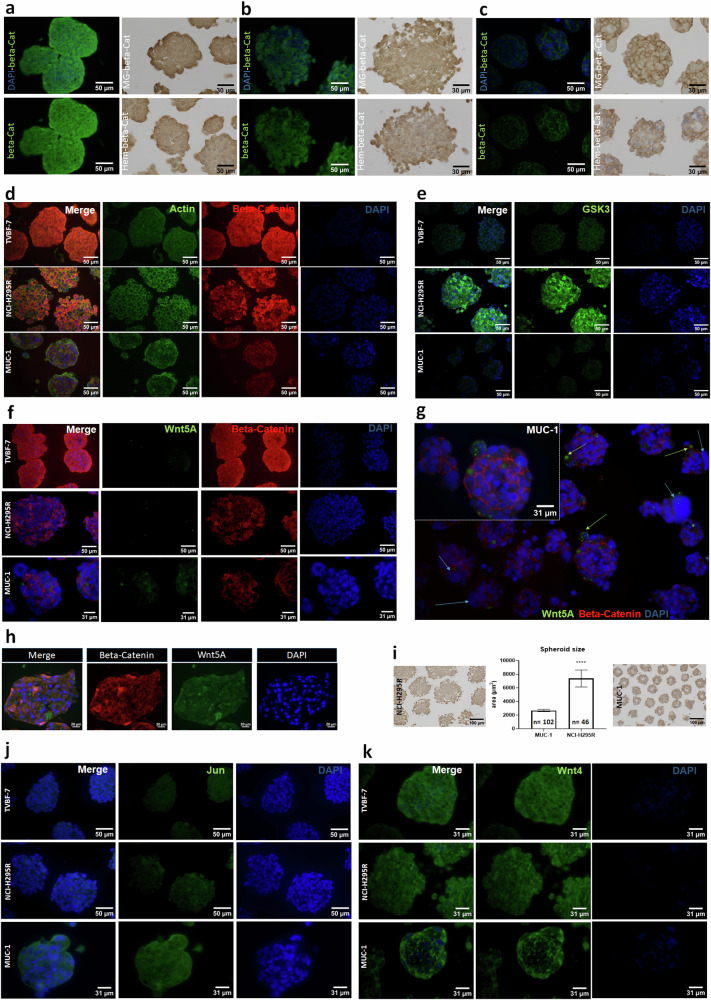
Cellular visualization of relevant canonical and noncanonical Wnt candidates in 3D models of ACC. **a**–**c** β-Catenin immunofluorescence (DAPI, Beta-Cat) and immunohistochemistry (Masson-Goldner/Beta-Cat, HE/Beta-Cat) in TVBF-7 (**a**) NCI-H295R (**b**) and MUC-1 (**c**) spheroids. **d**–**g** Immunofluorescence stainings of Actin–Beta-Cat–DAPI (**d**) GSK3–DAPI (**e**) Wnt5A–Beta-Cat–DAPI (**f**) in NCI-H295R, TVBF-7 and MUC-1 tumor spheroids. **g** Further selected pictures for Wnt5A-Beta-Cat-DAPI in MUC-1 tumor spheroids including a setting with lower magnification to provide an overview on different cellular localizations of relevant candidates over various spheroids. **h** Immunofluorescence for Beta-Cat and Wnt5A in primary-culture-derived tumor spheroids of a surgically resected primary ACC at an already metastasized stage. **i** Comparison of the total area of NCI-H295 and MUC-1 spheroids. **j**,**k** Immunofluorescence stainings of JUN–DAPI (**j**) and Wnt4–DAPI (**k**) in NCI-H295R, TVBF-7 and MUC-1 tumor spheroids.

Next, we performed comparative β-catenin immunofluorescence stainings in NCI-H295R, TVBF-7 and MUC-1 tumor spheroids. In accordance with the previously described overactivated canonical Wnt–β-catenin phenotype, we found cytoplasmic and strong nuclear β-catenin accumulation in NCI-H295R spheroids (Fig. 2b,d). However, for MUC-1, we detected very distinct membranous staining and enhancement of the closely linked actin filament network localized to the periphery of the spheroids (Fig. 2c,d). We also performed β-catenin immunohistochemistry on MUC-1 and identified cytoplasmic extensions surrounded by β-catenin-positive membranes (Fig. 2c). A precise pattern was not clearly distinguishable for TVBF-7 (Fig. 2a,d); instead, there was a mix of cytoplasmic, nuclear and membrane localization, as also depicted in the costaining with the nuclear lamina marker Lamin B1 in Fig. 4g. Notably, noncanonical (also known as β-catenin independent) Wnt-signaling pathways do not lead to nuclear shuttling of β-catenin. In the absence of canonical Wnt ligands, cytosolic β-catenin is either sequestered by the cytoskeleton as an interaction partner of cadherins and actin or actively degraded by the β-catenin destruction complex. Glycogen synthase-kinase 3 (GSK3) is an important regulator in this context and an essential part of the β-catenin destruction complex. GSK3 immunofluorescence in our models revealed strong, often spotted cytoplasmic staining for NCI-H295R spheroids. By contrast, the metastatic models showed both weaker and often very distinct nuclear accumulation of GSK3 (Fig. 2e).

Wnt4 and Wnt5A, which, based on the gene expression data, are specifically increased in TVBF-7 and MUC-1, are known to support context-dependent noncanonical signaling. To further explore noncanonical Wnt-signaling in our 3D models, we analyzed the cellular localization of noncanonical Wnt ligands. The gene expression experiments indicated elevated levels of the noncanonical Wnt ligand Wnt5A in the metastatic models compared with primary-culture-derived NCI-H295R (Fig. 1). Wnt5A protein levels are higher in the distant-metastatic MUC-1 cells compared with NCI-H295R and TVBF-7 cell lines (Fig. 2f). Interestingly in MUC-1, Wnt5A protein hotspots were often localized at the periphery of the tumor spheroid, which furthermore showed signs of potential budding or localized close to highly open chromatin (Fig. 2g). Notably, Wnt5a is a protein that has been shown to be part of the Wnt–PCP signaling pathway and to modulate PCP during directed cell migration. Next, as a further proof of concept, we established a primary culture of a resected tumor sample from a primary ACC at an advanced, highly metastatic disease stage (primary metastatic). We hypothesized that, under these conditions, the increased appearance of Wnt5A is also likely. In fact, in this model, we detected the highest Wnt5A abundance among all models (Fig. 2h).

**Fig. 3:**
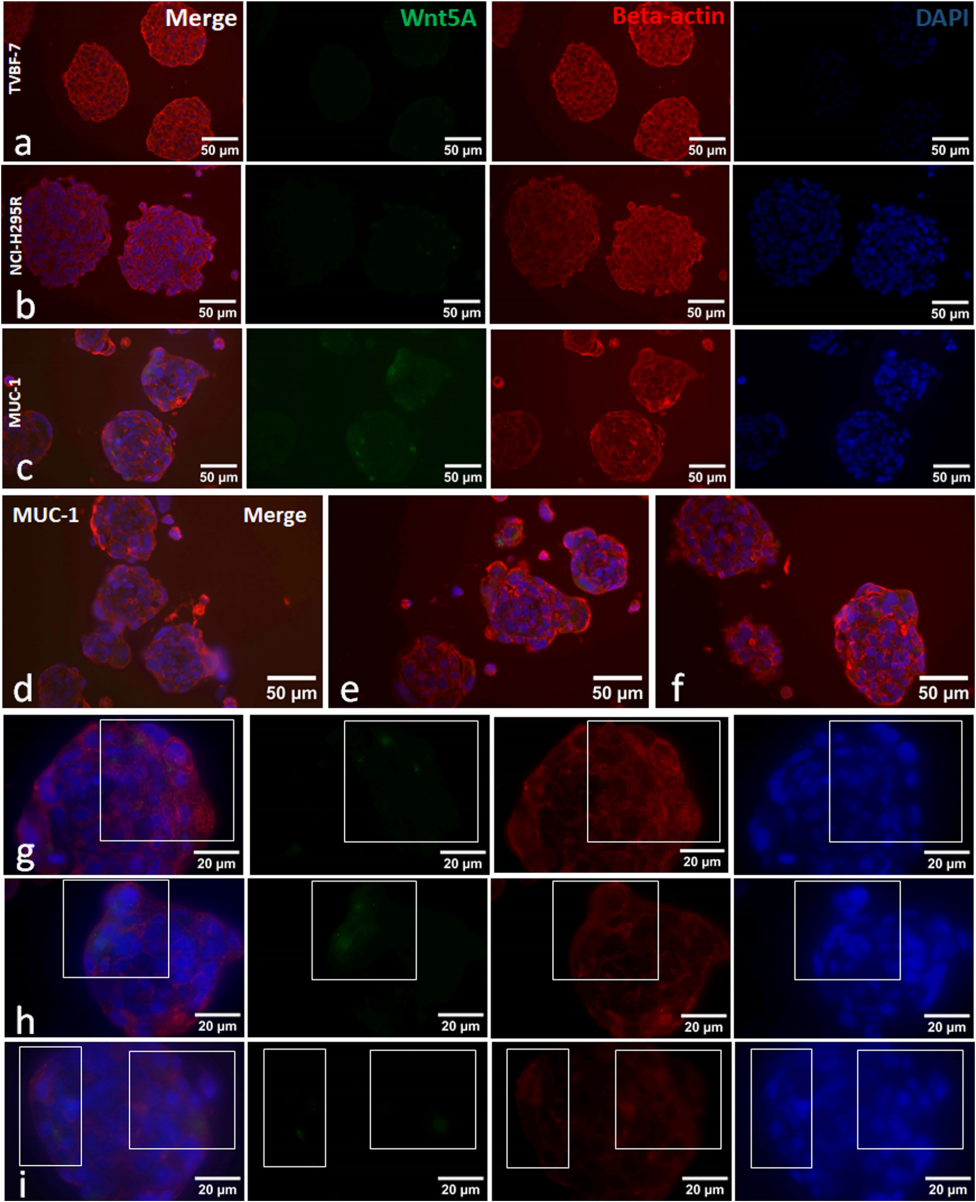
Microscopic visualization of Wnt5A and beta-actin in 3D-models of ACC. **a**–**i** Costaining of Wnt5A, beta-actin and DAPI in TVBF-7 (**a**) NCI-H295R (**b**) and MUC-1 (**c**) spheroids. Further selected pictures including high magnifications for MUC-1 (**d**–**i**).

Beside regulating rearrangements of the actin network, which were indeed found to be concentrated at the spheroid periphery for MUC-1 (Fig. 3), the Wnt–PCP signaling pathway controls tissue polarity and cell movements through the activation of specific signaling cascades including JNK. In accordance, we detected enhanced c-Jun stainings in MUC-1 tumor spheroids (Fig. 2j). Furthermore, initial gene expression data indicated a gradient in Wnt4 expression with regard to the models. However, this could not be confirmed at the protein level because all three models demonstrated comparable protein abundance of Wnt4 (Fig. 2k). Interestingly, in MUC-1, this protein was again distinctly located at the spheroid periphery.

#### Cellular localization of EMT and cytokeletal markers

**Fig. 4:**
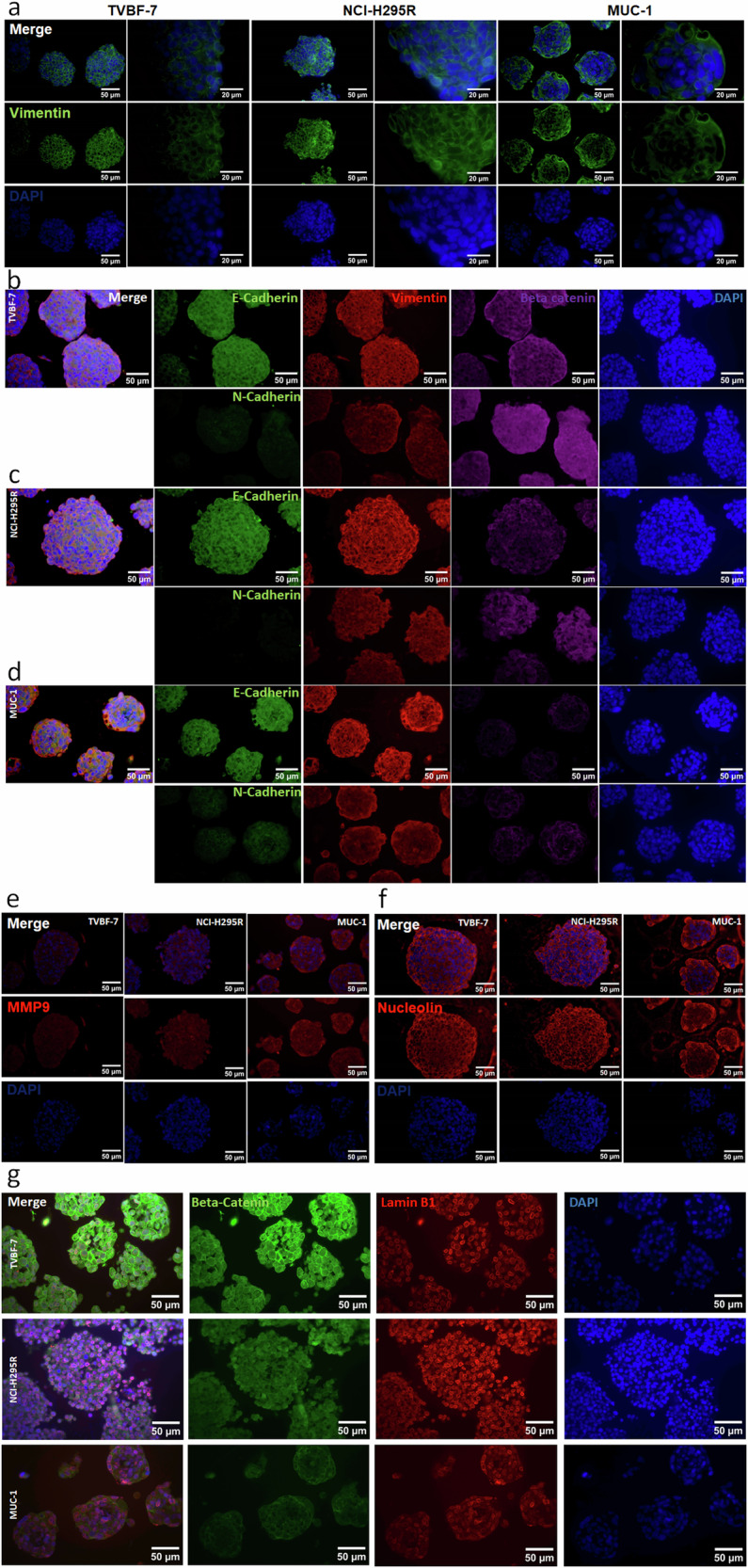
Localization of cytoplasmic and nuclear markers in TVBF-7, NCI-H295 and MUC-1 spheroids. **a** Cellular localization of vimentin in all three models. **b**–**d** The localization of E-cadherin and N-cadherin in TVBF-7 (**b**) NCI-H295R (**c**) and MUC-1 (**d**) spheroids. **e**–**g** The cellular localization of MMP9 (**e**) Nucleolin (**f**) and Lamin B1–β-catenin–DAPI (**g**) in tumor spheroids of all three models. Scale bar, 50 μm.

While we repeatedly observed continuous spheroid growth and increased diameter of NCI-H295R spheroids in our experiments, MUC-1 often appears to actively remodel spheroid growth down to highly compact sizes and shapes that often include celom-like structures (Fig. 2i). Notably, Wnt–PCP signaling is known to enable cells within a cellular network to receive positional information, to undergo lateral movements and to coordinate such movements with neighboring cells. These capabilities in the context of cancer are of high relevance regarding EMT and metastasization. Accordingly, we next investigated known features of such processes in the different models (Fig. 4a). We started with the intermediate filament vimentin, as intermediate filaments are critical components of the cytoskeleton and vimentin is a well-known marker in the context of EMT and metastasization. All three models expressed vimentin, but the pattern clearly differed for MUC-1, where the intermediate filament seems to fade in the spheroid core while in parallel appears intensified at the spheroid periphery (Fig. 4a). EMT is often described as hybrid staged between E-cadherin and N-cadherin, referred to as epithelial–mesenchymal plasticity, with increasing levels of N-Cadherin. Fittingly, we detected expression of N-Cadherin in both metastatic models, with the highest levels observed in MUC-1 (Fig. 4b–d). Triple E-cadherin–β-catenin–vimentin stainings once again reveal impressively enhanced vimentin signals in the spheroid periphery of MUC-1 (Fig. 4d).

The cytoskeleton is physically connected to the nuclear lamina, a network of polymerized A- and B-type lamins, between the nuclear envelope and chromatin. Further interesting markers in this specific context include Lamin B1 and Nucleolin, as well as MMP9 because it is frequently involved in the degradation of extracellular matrix (ECM) components. Consistent with our previous observations, we again detected enhanced MMP9 hotspots in the periphery of MUC-1 tumor spheroids (Fig. 4e). Both TVBF-7 and MUC-1 showed tendencies for comparably higher staining intensity for Nucleolin in the spheroid periphery, with maximum lateral levels in MUC-1 (Fig. 4f). Differential regulation of Lamin B1 was also observed, with a notable reduction and loss of distinct nuclear organization, as the protein faded from primary to distant-metastatic-derived models (Fig. 4g).

#### Gene expression and cellular localization of stemness markers

**Fig. 5:**
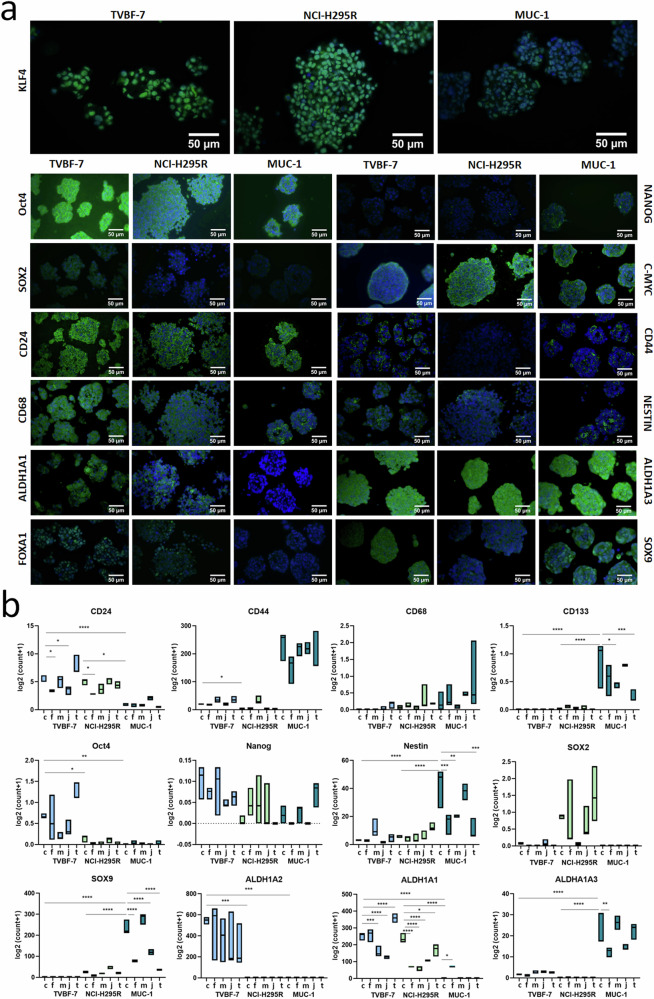
Expression and cellular localization of stem cell markers in TVBF-7, NCI-H295R and MUC-1 spheroids. **a** Immunofluorescence for KLF4, Oct4, Nanog, Sox2, c-Myc, CD24, CD44, CD68, Nestin, ALDH1A1, ALDH1A3, Foxa1 and Sox9 in TVBF-7, NCI-H295R and MUC-1 spheroids, presented as single stainings together with DAPI in each model. Scale bar, 50 μm. **b** Gene expression analysis of the various stem cell markers under control and treated conditions in monolayer cultures from all three cell models. Drug treatments from left to right: control (C), forskolin (F), mitotane (M), JQ1 (J) and THZ-1 (T).

Cellular plasticity, including EMT, contributes to pathological conditions and malignancies by providing cells with new phenotypic and functional characteristics beyond their originally assumed distinct cell states. Such features are often known to be related to stemness. Appropriate markers, including c-Myc, Sox9 and CD44 among others, are often downstream or interacting with Wnt-signaling pathways and also linked to HOX gene expression. Unfortunately, not much is known about stem cell markers in ACC. Thus, as a next step, we investigated the presence of model-specific stem cell patterns and the modulations of relevant markers under the same conditions as in Fig. 1. We started with a thorough characterization of the cell-fate-modulating Yamanaka factors (KLF4, Oct4, Nanog, SOX2 and C-Myc) and other well-known stem cell markers such as CD24, CD44, CD68, Nestin, ALDH1A1, ALDH1A3, FOXA1 and SOX9. The models demonstrated model-specific stem cell marker abundances (Fig. 5a) and expressions (Fig. 5b). Moreover, the important markers strongly oscillated under the same factors previously shown to modulate the main ACC cluster types (Fig. 5b).

#### Gene expression and modulation of Hox clusters

**Fig. 6:**
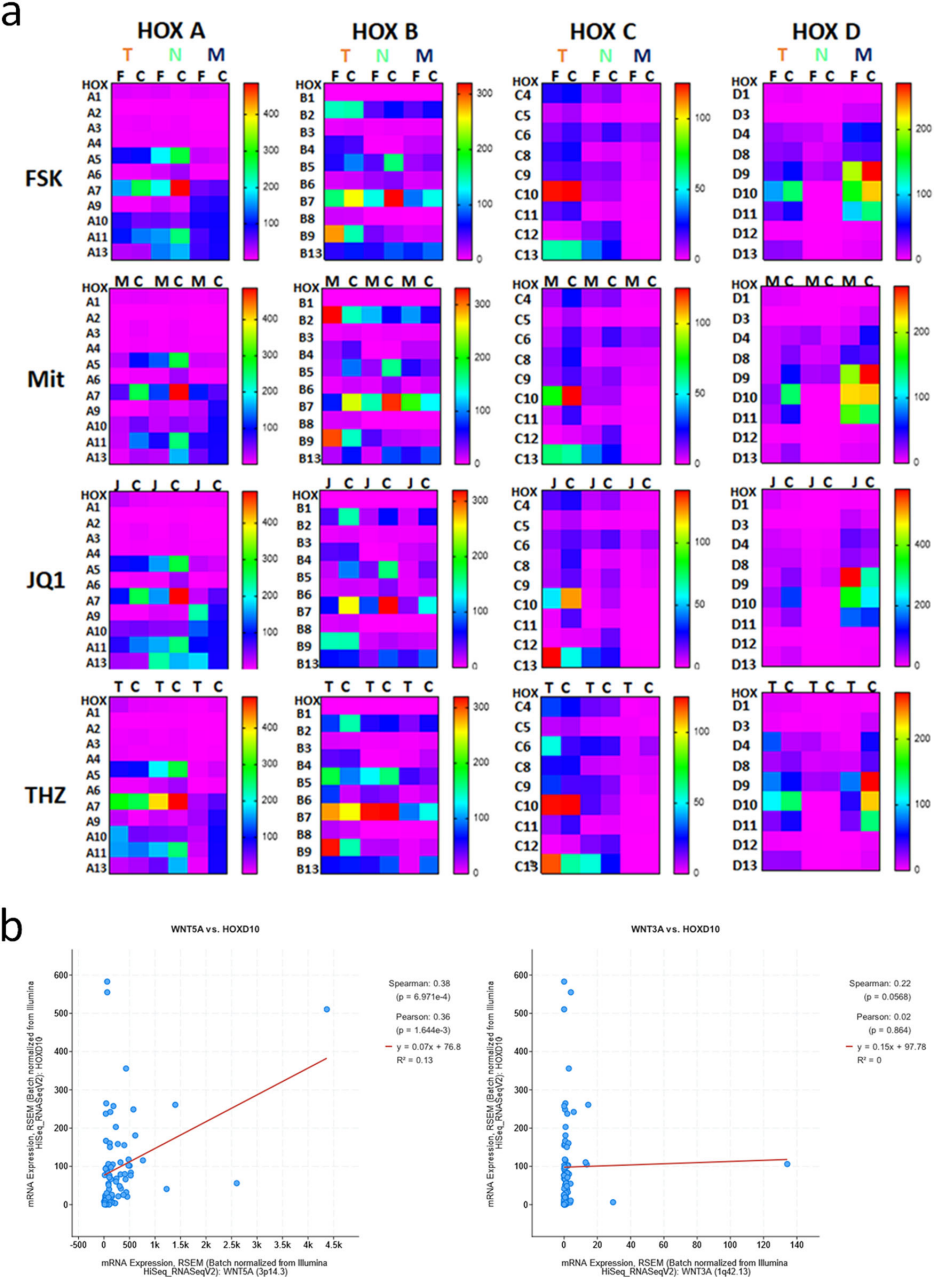
Analysis of HOX gene expression in preclincial models of ACC and in clinically relevant datasets from TCGA. **a** HOX gene expression by cluster in TVBF-7 (T), NCI-H295R (N) and MUC-1 (M) monolayer cultures under control (C) or treated conditions (*n* = 3): forskolin (FSK), mitotane (Mit), JQ1 and THZ-1 (THZ). The heat map represents the highest expression in red and lowest expression in pink. **b** Clinical correlation between the expression of Wnt5A and HOXD10, and Wnt3A and HOXD10, from patient datasets of TCGA.

Lastly, we analyzed the HOX family of transcription factors belonging to highly conserved gene clusters that determine cell fate and regulate Wnt signaling as well as stemness. Human Hox genes exist in four clusters and contain a DNA-binding homeobox region. Dysregulation of HOX transcription factors in numerous cancers supports tumorigenesis and cancer progression. Thus, to understand their contribution to ACC, we analyzed next all Hox genes (Fig. 6a). Interestingly, HOXA5/7 and HOXB5/7 coding regions were expressed and regulated in a model-dependent (SF-1 dosage) and drug-dependent (by modulating SF-1 dosage) manner. In addition, we found that the HOXD10 gene was exclusively expressed in TVBF-7 and MUC-1, with high expression of HOXD9 and HOXD11 in MUC-1, and HOXC10 in TVBF-7 spheroids. Interestingly, expression of HOXD10 also significantly correlated with Wnt5A, but not Wnt3A, expression in clinical patient samples as shown in TCGA (Fig. 6b).

## Discussion

Networks such as the Wnt pathways are fundamental not only during embryogenesis and for regenerative capacities of tissues but also in cancer initiation and progression. The upregulation or overactivation of the canonical Wnt pathway drives the proliferation of numerous tumors, including ACC, while its downregulation shifts the balance from tumor growth toward cellular differentiation^18^. Moreover, noncanonical pathways have been consistently associated with cell polarization and migration, linking them to invasive and metastatic processes in pathological conditions^13^. However, while the canonical Wnt–β-catenin pathway has been extensively studied over the past decades, the relevance of noncanonical pathways remains even nowadays elusive for ACC as well as other cancers. Moreover, culturing cell models in 3D space confers to them additional characteristics not present in cells grown in monolayers, closer to the in vivo state^29^. Notably, also the β-catenin stainings of NCI-H295R and MUC-1 3D models closely resemble multidimensional patterns previously observed in appropriate xenografts in vivo^26^. Here, we show gene expression as well as protein localization for critical components of canonical and noncanonical Wnt signaling in 2D and 3D cultures of ACC primary tumor (NCI-H295R), local (TVBF-7) and distant metastatic (MUC-1) origin. We observe Wnt-hybrid stages and propose that noncanonical Wnt signaling and related cytoarchitectural rearrangements provide specific features to the metastatic models TVBF-7, MUC-1 and a primary culture of a metastatic primary ACC compared with the nonmetastatic primary ACC model NCI-H295R. However, these pathways are probably not mutually exclusive, as we detect dynamic hybrid stages. These patterns are consistent with the in vivo origin of the models because activation of noncanonical Wnt pathways is associated with polarity and migration, two characteristics of metastatic tumors^13^. For example, MUC-1 spheroids exhibited the lowest β-catenin abundance and a predominantly membranous β-catenin localization, rather than the cytosolic/nuclear localization typically seen in canonical or overactivated Wnt signaling. For TVBF-7, we detected, in addition to cytoplasmic and nuclear, also high membraneous staining intensities, as clearly demonstrated in costainings with Lamin B1. Notably, the observed pattern of β-catenin is consistent with the mutational status of the Wnt–β-catenin pathway in the three cell models. For NCI-H295R, an overactivating mutation in the CTNNB1 gene has been reported. TVBF-7 is characterized by a nonsense APC mutation upstream in the canonical Wnt pathway. Finally, while Wnt–β-catenin is wild type in MUC-1, there is, in parallel, comparably low canonical signaling activity, as reflected by the observed low expression of LEF-1, the downstream partner of β-catenin, in this model^30^ (Fig. 1b and Supplementary Fig. 2). We propose that, given the localization and amount of β-catenin present, this molecule might be functioning primarily as a cytoskeletal component at the membrane in MUC-1, as will be discussed further on. In parallel, MUC-1 displayed the high gene expression and protein abundance of noncanonical Wnt5A protein in the spheroid periphery in addition to the expression of other components of Wnt–PCP.

Substantial evidence implicates noncanonical Wnt pathways in polarity, motility and stemness in cancer^13^. Cell polarity and motility are processes that rely heavily on the composition of the cytoskeleton and the kinases and phosphatases that regulate the polymerization and breakdown of actin, intermediate filaments and microtubules. Expression of β-actin specifically drives migration, while loss of β-actin affects the expression of cell cycle and motility genes^31^. The intermediate filament vimentin is associated with aggressiveness and is involved in the migration and polarity of tumor cells^32^. In our ACC metastatic models, the pattern of β-actin expression was highly organized in TVBF-7 spheroids (similarly to NCI-H295R) but very diffuse in MUC-1 spheroids. While vimentin staining was detected in all three ACC models, peripheral cells of the metastatic models had higher intensities, with the most pronounced staining in MUC-1. In both cases, β-actin- and vimentin-overexpressing cells were located specifically at the periphery of MUC-1 spheroids, as opposed to the inner bulk of the spehroids. Given that dynamic actin and vimentin networks characterize the invadopodias of metastatic cells^24^, this suggests that specific cells in MUC-1 spheroids are primed for invasion. Such zones were not always present in TVBF-7 spheroids, consistent with their local metastatic origin, possibly because these cells have not yet acquired the necessary machinery to break through the basement membrane.

Alterations in the cytoskeleton are insufficient for polarization and migration and must be complemented by the reconfiguration of the ECM and nucleoskeleton. In fact, the ECM, cytoskeleton and nucleoskeleton form a bidirectional kinetic chain that control cellular stiffness, chromatin accessibility and gene expression^33^. For example, ECM stiffness regulates proliferation and cell polarity, while E-cadherin expression correlates with the rigidity of the extracellular milieu. Although mechanosensing mechanisms are incompletely understood, alterations in the physical properties of cells positively correlate with the ability of cancer cells to invade^34^. Therefore, we investigated candidate proteins from the ECM and nucleus in the three ACC models. Lamin B1 is an integral part of the nucleoskeleton and interacts with the epigenetic landscape of inactive chromatin to actively suppress gene transcription^35^. It functions as a tumor suppressor because loss of Lamin B1 is associated with enrichment of genes involved in EMT, growth and migration^36^. All three ACC models expressed Lamin B1, although at different intensities, with the highest in NCI-H295R and lowest in MUC-1 spheroids. Particularly in MUC-1 spheroids, Lamin B1 was decreased in the nucleus, consistent with loss of Lamin B1 in the metastatic progression.

As a protein that localizes to both the nucleus and the plasma membrane, Nucleolin is involved in the regulation of multiple processes, from transcription to proliferation, angiogenesis and stemness^37^. Nucleolin expression is upregulated in many cancers and is currently being targeted by therapeutics because its inhibition blocks tumor growth and vasculature and induces apoptosis^37,38^. In our models, Nucleolin is mostly localized to the cytosol and plasma membrane in TVBF-7 and NCI-H295R spheroids. However, in MUC-1, the staining is diffuse but again highly concentrated at the periphery of the spheroids. Interestingly, Nucleolin synergistically interacts with Wnt signaling because its overexpression stabilizes β-catenin while its inhibition blunts canonical Wnt–β-catenin signaling^39^. MUC-1 expresses the least amount of both β-catenin and Nucleolin, consistent with our observations that noncanonical Wnt signaling underlies its metastatic phenotype.

Next, we assessed ECM–cytoskeleton interactions in our ACC models. Bound β-catenin contributes to cell–cell adhesion and cell polarity by bridging the connection between cadherins and the actin cytoskeleton, while unbound β-catenin is continuously degraded by cytoplasmic GSK3 in the absence of Wnt signaling^40^. Therefore, fluctuations in β-catenin and GSK3 abundance and localization would impact structural integrity, cell stiffness and mechanotransduction irrespective of Wnt signaling. Interestingly, in contrast to β-catenin, GSK3 was distinctly nuclear in the metastatic models, consistent with implications of nuclear GSK3 in metastasis, drug resistance and poor patient survival^41^. These findings suggest that β-catenin might mainly act as a structural protein in MUC-1, consistent with the expression of noncanonical Wnt signaling components in this model. Interestingly, interactions of β-catenin and cadherins at adherent junctions are implicated in adhesion, tension, proliferation and migration^40^. Changes in the expression of cadherins in cancer cells, such as the downregulation of E-cadherins and upregulation of N-cadherins, are associated with EMT, decreased cell adhesion and invasiveness^42^. Although E-cadherins are not detected in tumor samples from patients with ACC, this epithelial marker is expressed in NCI-H295R cells, where it, along with N-cadherin, is regulated by β-catenin signaling^43^. Accordingly, we report expression of E-cadherin and N-cadherin in all three models, with the highest expression of N-cadherin in MUC-1 consistent with its metastatic origin. In addition to E-cadherin^40^, canonical and noncanonical Wnt signaling directly target transcription factors that favor the E-cadherin-to-N-cadherin switch and cell motility as well as some of the MMPs necessary to cleave E-cadherins^44,45^. While all three ACC models expressed MMP9, we detected the highest levels for MMP9 in MUC-1 specifically at the periphery of the spheroids.

A recurrent theme in MUC-1 spheroids is the specific localization of many proteins associated with aggressive tumors to the periphery of the spheroids at zones that resemble potential budding sites. Tumor buds, which are present in some tumors in vivo, are projections located on the outskirts of the primary tumor that eventually detach from the main cell mass^46^. They are composed of a small cluster of cells that might represent the basis for metastasis because their presence correlates with invasiveness, distant metastasis and increased tumor staging^47^. At the molecular level, tumor buds are characterized by high vimentin, low β-catenin and E-cadherin expression, and the presence of proteases^46,48^. All these parameters are consistent with the data we report from MUC-1 spheroids, namely the presence of decreased β-actin, vimentin, Nucleolin and MMP9 along with the presence of often highly open chromatin in zones that appear in the periphery of MUC-1 spheroids.

Unraveling the interplay between WNT signaling and its downstream stemness effectors is pivotal to understanding disease progression and developing new therapeutic treatments. Wnt signaling has been linked to stemness, proliferation and metastasis^13,18^, and its inhibition blocks metastasis^49^. The plasticity of cancer-stem-cells (CSCs) is well documented and contributes to the ability of the tumor to adapt and metastasize^50^. Here, we report that all three ACC models express multiple stemness markers at the transcriptional and protein levels, suggesting the presence of cancer stemness in these validated preclinical ACC models, which reflect relevant clinical cluster types^25^. This study characterizes a broad panel of stem cell markers in ACC models. Consistent with our preclinical data, a stemness signature has been recently bioinformatically predicted on the basis of RNA sequencing data and clinical characteristics for ACC downloaded from TCGA^51,52^. We demonstrate expression of the markers CD24, CD44, CD133 and ALDHA1A, which are well known to characterize cancer stem cells^53,54^. Interestingly, our models also stain positive for CD68, which is often implemented as a marker for infiltrating macrophages but which has also been identified in lymphoid cells, nonhematopoietic cells, mesenchymal stem cells and tumor cells^55^. Notably, the cellular function of CD68 remains elusive, although it has been suggested to play roles in peptide transport, antigen processing and as a scavenger receptor for oxidized low-density lipoprotein^55^. Of note, our models did not include settings with cocultured immune cells, suggesting that CD68 may play a distinct role within ACC tumor cells themselves. Furthermore, we show that the core reprogramming factors KLF4, Oct4 and cMyc (also known as the Yamanaka factors) are highly expressed in spheroids of all three models. These factors, first identified for their ability to convert somatic cells into induced pluripotent cells, dictate cell fate and correlate with poor survival in many tumors^53,54,56,57^. The downstream effectors of the Yamanaka factors, Nanog, FOXA1 and Nestin, were also induced in spheroids from all three models. Nanog, along with SHH, HOX and Wnt–β-catenin, is implicated in maintaining stemness^17,58^, while Nanog and FOXA1 are implicated in therapy-resistant cancer^59^. Interestingly, Oct4 directly interacts with Nucleolin and along with Nanog is overexpressed in cancer stem cells^60,61^, while stem cell marker CD133 is induced by Nucleolin^62^. Furthermore, Nestin expression associates with dedifferentiation, invasiveness and increased aggressiveness of tumors^63^. Importantly, the expression of many stem cell markers in our in vitro models were modulated by treatments, similarly to the regulation we described for the Wnt and SHH signaling pathways. For example, mitotane and THZ-1 treatments significantly reduced levels of Nestin and CD133, while forskolin significantly reduced ALDHA1A3 expression in MUC-1. The potential presence of stem cells in ACC models is consistent with their aggressive phenotype and their recurrence after surgical and pharmaceutical interventions. Because cancer-stem-cells both dedifferentiate and differentiate into multiple cell types, understanding their role in ACC and the drug-induced shifts in their phenotype could enhance the potential for successful treatment.

The HOX and Wnt pathways can act synergistically to promote invasiveness and metastasis^14^, and HOX expression correlates with increased proliferation, aggressive disease and poor survival of patients with ACC^17^. Although aberrant expression of HOX genes has been demonstrated in various tumors, their role as oncogenes versus tumor suppressors varies by tumor and tissue. Here, we show that all three ACC models express many of the HOX genes from all four clusters and that drug treatments cause dynamic shifts in their expression. Specific genes from the HOXC and HOXD clusters are highly expressed in the metastatic models (but not expressed in NCI-H295R) such as HOXC10/13 (TVBF-7), HOX D9/11 (MUC-1) and HOXD10 in both. Interestingly, HOXD10 is significantly expressed and modulated in the metastatic models, although there are reports that HOXD10 acts as a tumor suppressor^17^. However, metastases regulate their proliferative properties to restrict spheroid size, as we report here for MUC-1, suggesting that perhaps HOXD10 is acting in this capacity to preserve the integrity of TVBF-7 and MUC-1 spheroids. We speculate that the size and proliferative properties of metastasis might be linked to the activation of noncanonical Wnt pathways. Consistently, HOXD10 expression specifically and significantly correlates with Wnt5A in clinical patient samples from TCGA. In contrast to HOXD10, its paralog HOXC10 is highly expressed only in TVBF-7 where it is downregulated in response to mitotane and JQ1, underscoring again the importance of characterizing models of different metastatic origin. Furthermore, both these genes, in addition to several other Hox genes, including HOXB7–9, are known to regulate MMPs, vimentin, E-cadherin, N-cadherin and other EMT genes in migratory and metastatic cancer cells^17^. HOXB9 is also implicated in aggressive ACC^16^ and can act synergistically with Wnt signaling^14^.

Overall, our work sheds light on specific pathways involved in cell fate, invasion, stemness and most important related cytoskeletal rearrangements in 2D and 3D models of ACC. Our findings are particularly relevant as we provide not only specific gene expression patterns, but also related information about protein localizations in 3D spheroid cultures.

## Supplementary information

Supplementary Information
