# Supplementary material for: (Non)canonical Wnt signaling, cytoarchitecture and stemness: new insights from primary nonmetastatic, primary metastatic, regional and distant metastatic models of adrenocortical carcinoma
Source: Exp Mol Med. 2025 Nov 28;57(11):2657–70. doi: 10.1038/s12276-025-01507-z (PMC12686465; doi:10.1038/s12276-025-01507-z)
Supplement: Supplementary file 1 — Supplementary Information [file 12276_2025_1507_MOESM1_ESM.pdf]

Supplementary Fig. 1:

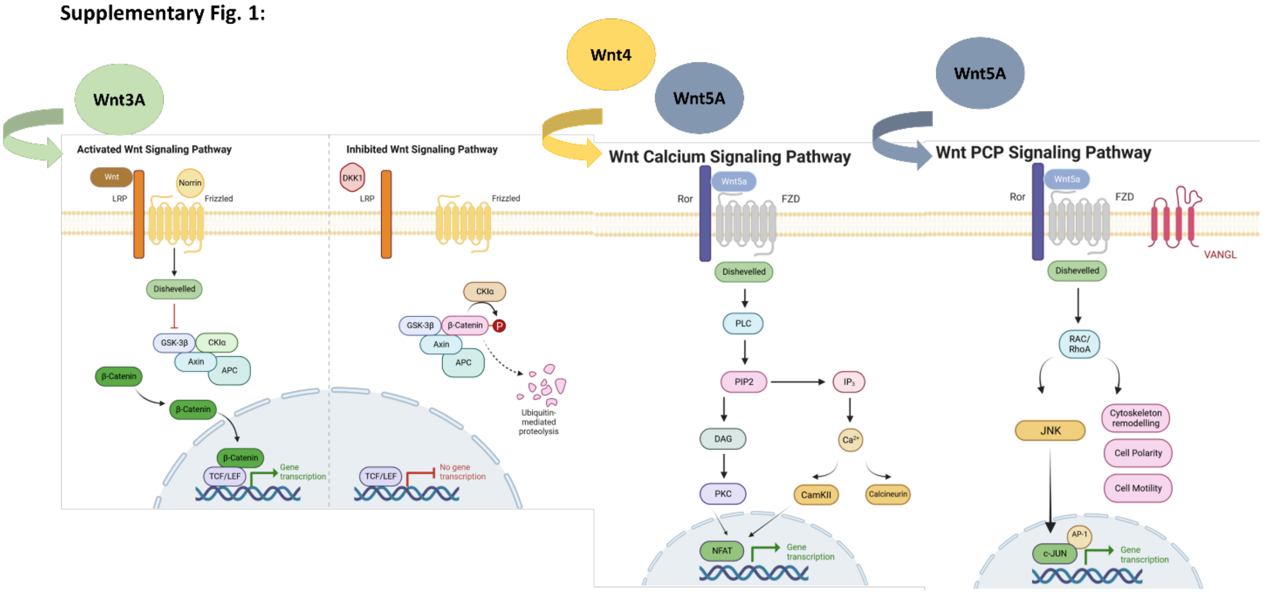

Supplementary Fig. 1: Cartoon depicting activated/inhibited canonical Wnt signaling and non-canonical pathways Wnt-Calcium and Wnt-PCP. Created with Biorender.

Supplementary Fig. 2:

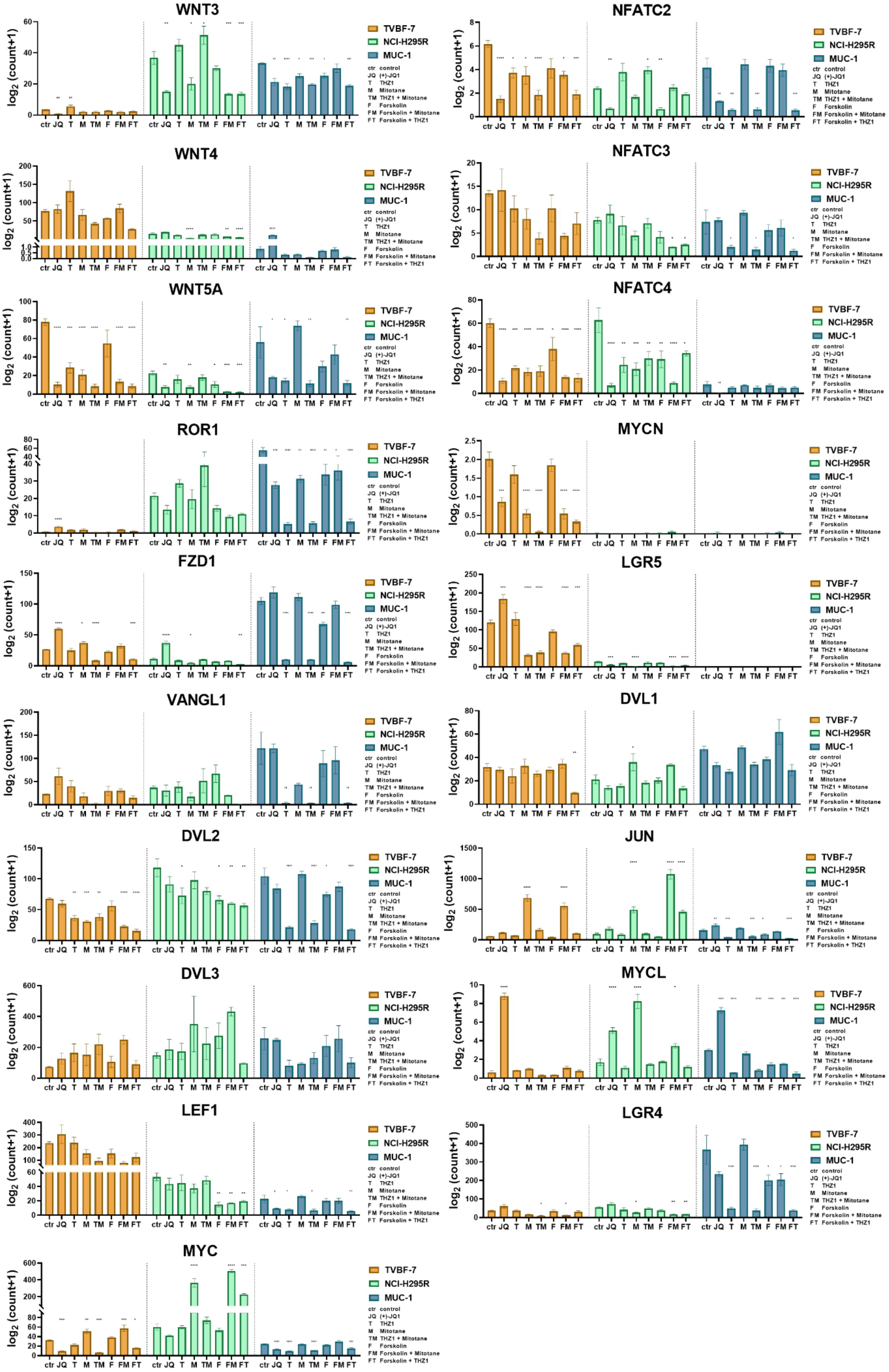

Supplementary Fig. 2: High magnification of the gene expression data presented in Fig. 1 of selected signaling components of Wnt-β-catenin, Wnt-$Ca2^{+}$, Wnt-PCP under different conditions and for th three different models TVBF-7, NCI-H295R and MUC-1. From left to right: untreated controls (C), JQ1 (J), THZ-1 (T), mitotane (M), THZ-mitotane (TM), forskolin (F), forskolin-mitotane (FM), forskolin-THZ1 (FT), (n=3).
